# Epigenetic mechanisms in aneurysm formation, growth, and rupture: A systematic review

**DOI:** 10.1177/15910199251391864

**Published:** 2025-12-04

**Authors:** Oleg Shekhtman, Irina-Mihaela Matache, Georgios S Sioutas, Sandeep Kandregula, Najib Muhammad, Ilayda Kayir, Michael Covell, Stephen Capone, Gennadii Piavchenko, Joshua S Catapano, Jan-Karl Burkhardt, Visish M Srinivasan

**Affiliations:** 1 189491 Department of Neurosurgery, 14640 Perelman School of Medicine, 6572 University of Pennsylvania, Philadelphia, PA, USA; 2 Department of Physiology, 87267 Carol Davila University of Medicine and Pharmacy, Bucharest, Romania; 3 52946 Bahcesehir University School of Medicine, Istanbul, Turkey; 4 12230 Georgetown University School of Medicine, Washington, DC, USA; 5 Department of Neurology, 145763 Virginia Tech Carilion School of Medicine, Roanoke, VA, USA; 6 Department of Human Anatomy and Histology, Sechenov University, Moscow, Russia

**Keywords:** Epigenetic, intracranial aneurysms, aneurysm formation, aneurysm inhibition, aneurysm rupture

## Abstract

**Introduction:**

Intracranial aneurysms (IAs) affect approximately 3.2% of the global population, and their rupture leading to subarachnoid hemorrhage remains a significant cause of morbidity and mortality despite therapeutic advancements. While genetic factors have been implicated in IA pathogenesis, they account for only about 41% of heritability, suggesting that other mechanisms—particularly epigenetic modifications—may play a critical role. Epigenetic processes such as DNA methylation, histone modification, and non-coding RNA regulation have been shown to mediate gene–environment interactions, influencing endothelial function and vascular remodeling. This systematic review aims to synthesize the current literature on epigenetic mechanisms implicated in IA development, progression, and rupture.

**Methods:**

The review was conducted in accordance with PRISMA guidelines, including both in vitro and in vivo studies available in PubMed up to November 2023. A total of 1019 studies were screened, resulting in 77 eligible full-text articles for data extraction.

**Results:**

The most frequently studied mechanisms were microRNAs (59.7%), DNA/RNA methylation (20.8%), circular RNAs (7.8%), long non-coding RNAs (6.5%), and histone modifications (5.2%). Notably, only three overlapping epigenetic targets were identified across studies, underscoring the field's methodological heterogeneity and lack of standardization. These individual epigenetic pathways are further examined in detail within the Discussion section.

**Conclusion:**

These findings underscore the emerging role of epigenetic research in elucidating novel pathways of intracranial aneurysm pathogenesis, while the limited reproducibility across studies highlights the need for standardized methodologies and larger, more diverse cohorts. Epigenetic regulation remains a promising target for future genetic and therapeutic investigations.

## Introduction

Systematic reviews and meta-analyses suggest that the global prevalence of unruptured intracranial aneurysms (IAs) is around 3.2%.^1^ Despite recent treatment advancements, spontaneous subarachnoid hemorrhage (SAH) resulting from ruptured IAs continues to have a poor prognosis, with disability and mortality rates still as high as 30–40%.^2^ Therefore, further advances in understanding the pathogenesis of IAs are necessary for developing effective diagnostic and prevention strategies for aneurysms.

Genetic factors play a crucial role in the development of IA and aSAH, with the likelihood of these conditions increasing based on the number of first-degree relatives affected in a family.^3^ Individuals with hereditary disorders such as Ehlers–Danlos syndrome, Marfan syndrome, and neurofibromatosis-1 have significantly higher rates of IAs (10–20%) and aneurysm rupture (8–25%) compared to the general population.^4,5^ Large-scale genome-wide association studies (GWAS) conducted mainly on Dutch, Finnish, and Japanese populations have identified several single nucleotide polymorphisms (SNPs) that are more common among those with IAs.^6–8^ Despite this, genetic variations account for only 41% of the heritability of the disease, leaving a large proportion of the risk unexplained.^9^

As a result, environmental factors have been suggested as contributors to the increased familial risk, possibly through epigenetic modifications.^10^ Epigenetic mechanisms, including DNA methylation and non-coding RNAs, have been shown to regulate endothelial cell proliferation, apoptosis, migration, and vascular malformation, thus being associated with formation of intracranial arterial-venous malformation.^11^ Interestingly, many IA-risk loci are located in functional, noncoding regions, indicating that genetic risk may influence regulatory elements that affect gene expression rather than altering the structure of the gene product itself.^5,12^ Epigenetic mechanisms mediate the interaction between genes and the environment, thereby impacting susceptibility to various complex diseases. This critical role in regulating gene expression has led to a growing focus in recent years on the epigenetic regulation of IA growth and rupture as a potential target for treatment.^13^

In this systematic review, we aimed to comprehensively review the current body of literature on key epigenetic mechanisms to identify prevailing trends and highlight the most promising directions for future research.

## Materials and methods

### Review design

**Figure 1.**
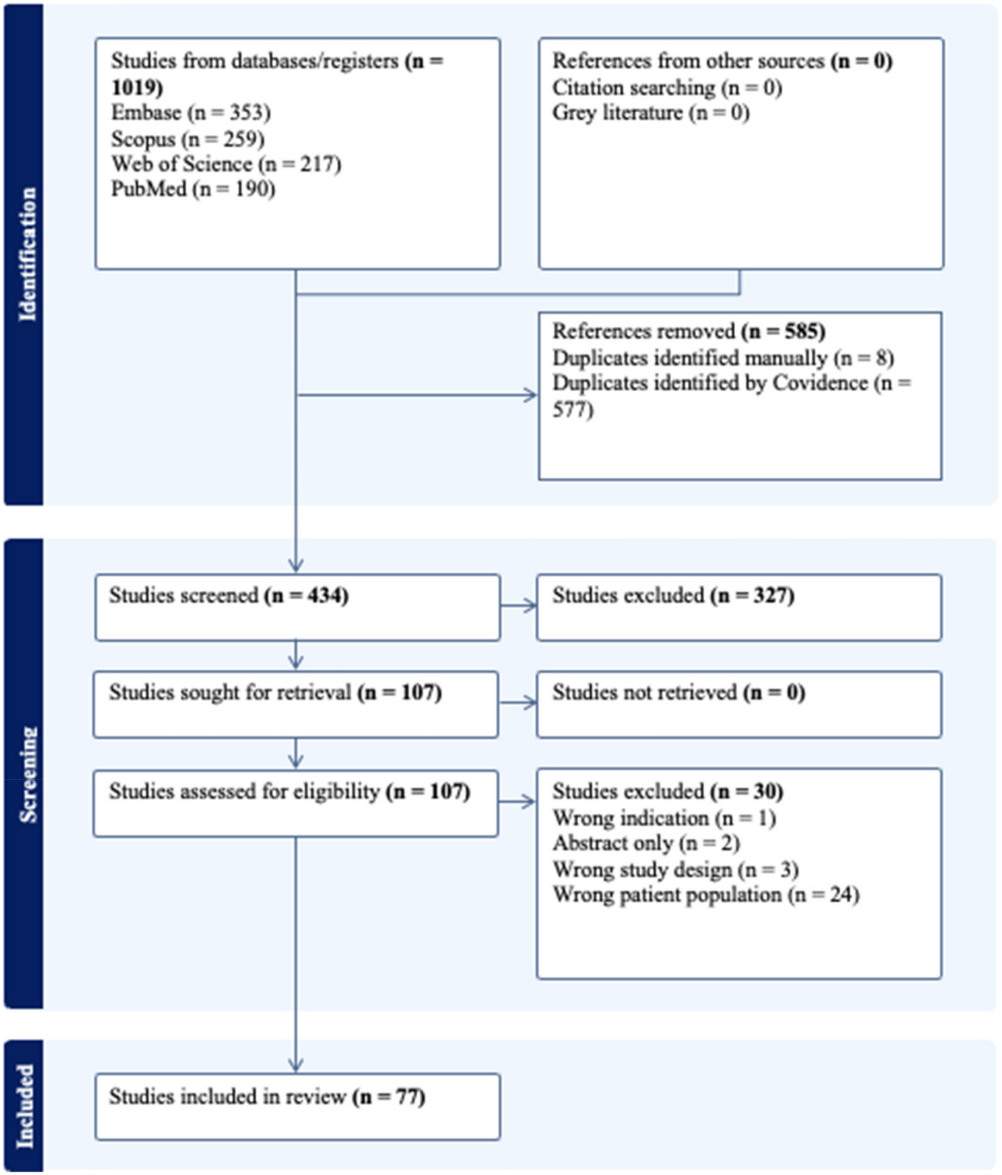
PRISMA flowchart depicting the workflow of the study selection process, in accordance with the PRISMA guidelines.

Review of the literature was conducted in accordance with the Preferred Reporting Items for Systematic Reviews and Meta-Analyses (PRISMA) guidelines. The systematic search encompassed studies that examined various mechanisms of epigenetic regulation in the development, progression, or rupture of intracranial aneurysms. Both in vitro and in vivo studies were included regardless of the age of participants. Only English-language full-text papers were considered. The complete workflow is presented as a diagram in Figure 1.

### Search strategy

Keywords were identified using the MeSH (Medical Subject Headings), the NLM-controlled thesaurus used for indexing articles for PubMed. The keywords employed for the database search were “intracranial aneurysms” combined with the following epigenetic mechanisms: histone modification, histone acetylation, histone methylation, histone phosphorylation, DNA methylation, DNA acetylation, DNA phosphorylation, microRNA, circular RNA, long noncoding RNA, and small interfering RNAs.

### Study selection

Studies published in PubMed by the end of 2023 were included. Additionally, a manual search was conducted concurrently to ensure no relevant documents were omitted. Titles and abstracts were thoroughly screened by two reviewers (OS and GSS), resulting in the selection of full-text articles for review, as detailed in the PRISMA flowchart. The review process utilized the Covidence app for guidance. Four reviewers (SC, MC, IMM, and GSS) screened the full articles and reached a consensus on final inclusion, followed by data extraction. Consensus on the inclusion or exclusion of studies was determined through a vote by a panel of three researchers. Studies that received a minimum of two positive votes were included for data extraction. The extracted data encompassed the authors, year of publication, country of origin, study model and number of samples, main epigenetic mechanism studied, methods, and key findings.

## Results

A total of 1019 studies were screened, and all references retrieved were imported into the Covidence application. After removing duplicates, 434 journal articles were included in the study. Based on the title and abstract screening, and consensus between two blinded reviewers (MC and SC), 327 studies were excluded. Twenty-four papers were excluded due to having the wrong patient population, three due to the wrong study design, two had no full text version, and one due to the wrong indication. In the second round of review, which involved full-text assessment, 77 articles were selected for further evaluation. The breakdown by key epigenetic targets is shown in Table 1.

**Table 1.** Breakdown of papers included by key epigenetic targets.

Epigenetic target	*N* (%) of papers
MicroRNA (mRNA)	46 (59.7)
DNA or RNA methylation (DNAm, RNAm)	16 (20.8)
Circular RNA (circRNA)	6 (7.8)
Long non-coding RNA (lncRNAs)	5 (6.5)
Histone modification	4 (5.2)

Other potential targets were not found in the searched studies. The distribution by country is summarized in Table 2.

**Table 2.** Distribution of epigenetic research papers on the subject by country.

Country of origin	*N* (%) of papers	Country of origin	*N* (%) of papers
China	64 (83.1)	Saudi Arabia	1 (1.3)
India	4 (5.2)	Turkey	1 (1.3)
USA	3 (3.9)	Brazil	1 (1.3)
Netherlands	2 (2.6)	Total	77 (100)

**Figure 2.**
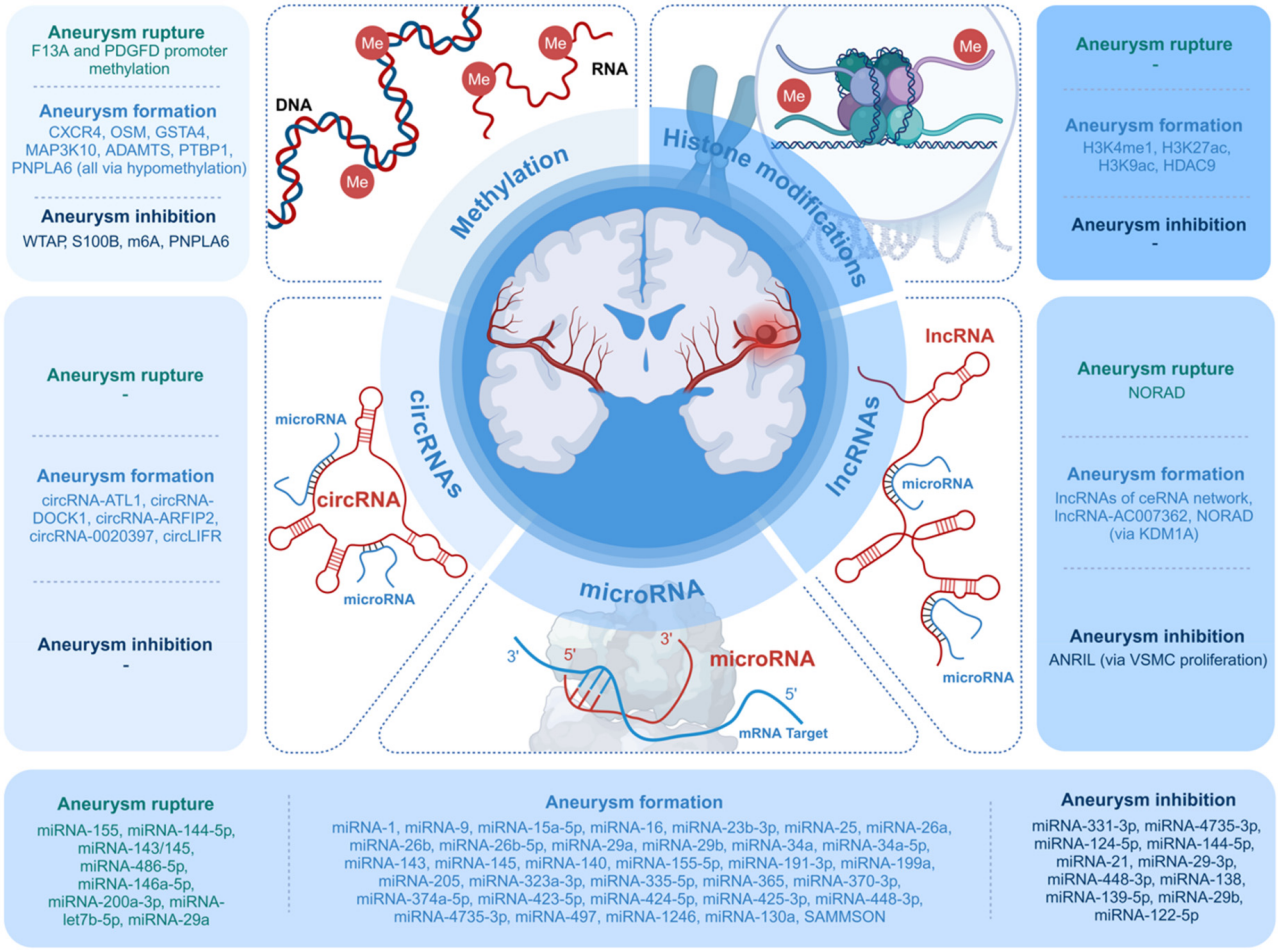
Epigenetic mechanisms associated with aneurysm formation, inhibition, and rupture.

VSMC: vascular smooth muscle cell; NORAD: non-coding RNA activated by DNA damage; SAMMSON: survival associated mitochondrial melanoma-specific oncogenic non-coding RNA.

As most papers aimed at clinically applicable goals (identifying mechanisms involved in aneurysm development or risk of subarachnoid hemorrhage [SAH]), we categorized all studied epigenetic targets as associated with aneurysm formation, rupture, or prevention (“protective”). This result is shown in Figure 2.

A more detailed overview of the papers, including author, year, study model, number of samples, epigenetic target, and main findings, can be found in Tables 3–5.

**Table 3.** Overview of studies focusing on microRNA as the primary epigenetic target.

Author, year	Study model	*N* of samples	Epigenetic target	Main findings
Al-Jehani, 2023^14^	Human blood	10 ruptured-IA patients 7 unruptured-IA patients	miRNA-16, miRNA-143, miRNA-200	miRNA-16, miRNA-143, and miRNA-200 were significantly upregulated in IA patients, with miRNA-200 doubled in those with single IA and miRNA-143 increased in ruptured IA.
Boga, 2023^15^	Human IA tissue	50 patients	miRNA-26a, miRNA-29a, and miRNA-448-3p	Expression levels of miRNA-26a, miRNA-29a, and miRNA-448-3p were significantly increased in aneurysm tissues compared with normal vascular tissues.
Fan, 2020^16^	Human IA tissue, rat IA model.	96 IA patients 51 Sprague Dawley rats with IA model	miRNA-331-3p	miRNA-331-3p inhibited the formation and progression of IAs by downregulating expression of the tumor necrosis factor alpha (TNF-α) and CD14, thereby maintaining the contractile phenotype of VSMC and reducing VSMC apoptosis.
Liao, 2020^17^	Human plasma	34 unruptured-IA patients 47 ruptured-IA patients	Exosomal miRNAs	Exosomal miRNA-145-5p and miRNA-29a-3p were significantly upregulated in IA patients compared to healthy controls
Feng, 2019^18^	Human IA tissue, rat IA model	26 IA patients 24 Sprague-Dawley rats for IA modeling	miRNA-155-5p	Tumor-associated macrophage (TAM)-derived exosomal miRNA-155-5p promoted IA formation and progression by downregulating expression of *Gremlin 1* (*GREM1*) gene, which enhances SMC proliferation and migration, and increased TAM activation and infiltration.
Gao, 2019^19^	Human IA tissue, human umbilical artery SMC, human VSMC	30 unruptured-IA patients 30 ruptured-IA patients	miRNA-4735-3p	Downregulation of miRNA-4735-3p in IA tissues increased hypoxia inducible factor-1 (HIF-1) expression, promoting autophagy-mediated proliferation and migration of VSMCs. Overexpression of miRNA-4735-3p suppressed *HIF-1* gene expression, reducing VSMC proliferation and migration.
Yang, 2019^20^	Human IA tissue, human blood	48 ruptured-IA patients 46 unruptured-IA patients	miRNA-155	Single-nucleotide polymorphism (SNP) rs767649 in the promoter region of miRNA-155 reduced its transcription activity, leading to lower miRNA-155 levels. Reduced miRNA-155 increased *MMP-2* gene expression, which is associated with a higher risk of IA rupture, particularly in patients with the TT genotype.
Zhong, 2019^21^	Human blood, human IA tissue	91 unruptured-IA patients	miRNA-205	miRNA-205 was significantly upregulated in IA patients. Overexpression of miRNA-205 reduces VSMC viability and downregulates Hepatocyte Growth Factor (*HGF*) gene expression, indicating its role in IA pathogenesis and potential as a diagnostic biomarker.
Wang RK, 2021^22^	Human umbilical vein EC	30 patients	miRNA-124-5p	miRNA-124-5p expression was lower in blood of IA patients and interleukin 1β (IL-1β)-stimulated human umbilical vein ECs. miRNA-124-5p inhibited migration and invasion in human umbilical vein EC, the release of inflammatory factors. Its overexpression suppresses the expression of the *FOXO1* gene member of the FOXO family of transcription factors and tumor suppressors, while its inhibition promoted these effects, which *FOXO1* knockdown can reduce.
Zheng, 2021^23^	Human serum, human arterial SMC	100 unruptured-IA patients 100 ruptured-IA patients	miRNA-513b-5p	In ruptured and unruptured IAs, miRNA-513b-5p, interleukin (IL)-6, and TIMP4 levels were decreased, while COL1A1, COL1A2, tumor necrosis factor alpha (TNF-α), IL-1β, MMP2, MMP3, and MMP9 expression levels were increased. miRNA-513b-5p targets collagen-type I alpha 1 chain (*COL1A1*) and *COL1A2*, regulating pathways that enhance cell death and apoptosis. Overexpression of miRNA-513b-5p or silencing *COL1A1/2* inhibits TNF-α-induced cell proliferation, promotes cell death, and affects collagen and MMP expression.
Yang, 2021^24^	Human plasma, human vascular EC, rat IA model	51 unruptured-IA patients 33 ruptured-IA patients	miRNA-144-5p and its target gene PTEN	miRNA-144-5p levels were lower in serum exosomes from unruptured and ruptured IA patients. Overexpression of miRNA-144-5p in mesenchymal stem cells (MSC)-derived exosomes improved vascular EC viability, inhibited proliferation, and decreased the MMP-9, proliferating cell nuclear antigen (PCNA), and osteopontin (OPN) protein levels. In the rat model, miRNA-144-5p overexpression in exosomes reduced IA progression, apoptosis, and expressions of matrix MMP-9, PCNA, and OPN.
Supriya, 2022^25^	Human IA tissue	29 ruptured-IA patients	Multiple miRNAs	In aneurysmal tissues, miRNA-26b, miRNA-199a, miRNA-497, and miRNA-365 were significantly decreased. These miRNAs target genes in the transforming growth factor-beta (TGF-β) and mitogen-activated protein kinases (MAPK) pathways, affecting inflammation, extracellular matrix degradation, VSMC function, and apoptosis. They showed a significant negative correlation with mRNA levels of TGF-β1, TGF-β2, SMAD family member 2 (SMAD2), SMAD family member 4 (SMAD4), MAPK1, and MAPK3.
Yuan, 2021^26^	Human IA tissue, human VSMC, human serum	20 patients	miRNA-34a targeting CXCR3 and MMP-2	In IA patients, downregulated miRNA-34a inhibits VSMC proliferation and migration by targeting CXCR3, which promotes MMP-2 expression through shared miRNA-34a targets, and its overexpression reverses CXCR3's effects on these processes.
Li et al., 2021^27^	Human VSMC, human umbilical artery SMC	9000 cells per well each	miRNA-29b	miRNA-29b downregulated transforming growth factor-beta (TGF-β) expression by targeting its mRNA's 3'-UTR, modulating cell migration and proliferation through the TGF-β1/Smad3 signaling pathway.
Bi et al., 2021^28^	GEO	49 abdominal, 44 intracranial, and 12 thoracic aneurysm datasets	miRNA, miRNA-connected genes	*CCR7*, tumor necrosis factor (*TNF*), and *CXCR4* were the most connected genes in miRNA-hub genes network; hsa-miRNA-26b-5p and hsa-miRNA-335-5p were the most connected miRNAs. No results attributed solely to IA were reported.
Ma et al., 2020^29^	Human IA tissue, human umbilical artery SMC	18 patients	miRNA-29b, cell division	miRNA-29-3p inhibited cell proliferation and mobility by directly targeting *CDC7*, which could be a potential therapeutic target for vascular dysfunction.
Jin et al., 2020^30^	Human plasma	16 patients	miRNA-21	miRNA-21 had a protective effect for vascular wall against remodeling and warning function for IA rupture
Zhao et al., 2020^31^	GEO	–	miRNA	Nodes and edges analyses revealed enrichment in cell cycle, proliferation, and PI3K/Akt signaling pathways, while hsa-miRNA-191-3p,hsa-miRNA-423-5p, hsa-miRNA-424-5p, and hsa-miRNA-425-3p were proven to be valuable IA predictors.
Supriya et al., 2020^32^	Human plasma	20 patients	miRNA	Three upregulated (miRNA-15a-5p, miRNA-34a-5p, miRNA-374a-5p) and five downregulated miRNAs (miRNA-146a-5p, miRNA-376c-3p, miRNA-18b-5p, miRNA-24-3p, miRNA-27b-3p) could distinguish patients with aSAH from healthy controls
Yang et al., 2020^33^	Human plasma	102 patients	miRNA-126	miRNA-126 may serve as a potential diagnostic and predictive indicator for IA occurrence and rupture.
Zhang et al., 2018^34^	Sprague-Dawley rat model	–	miRNA-448-3p	miRNA-448-3p plays the inhibitory role in IA progression through downregulation of macrophage-mediated inflammation
Xu et al., 2018^35^	Human IA tissue	30 patients	miRNA-143/145, KLF5	Overexpression of the miRNA-143/145 cluster inhibited VSMC proliferation and migration, increased contractile protein expression, and decreased MMP-2 and MMP-3, while KLF5 overexpression had the opposite effect.
Feng et al., 2018^36^	Human plasma	24 patients	miRNA-143/145	Lower plasma miRNA-143/145 levels are potentially associated with IA formation, while higher serum MMP-9 levels could be associated with IA rupture.
Lopes et al., 2018^37^	Human plasma	33 patients	miRNA	Eight miRNAs were differentially expressed: three up-regulated and five down-regulated. miRNA-486-5p was the most abundant and linked to poor neurological status.
Guo et al., 2018^38^	Human VSMC	32 patients	miRNA-23b-3p	Cells transfected with miRNA-23b-3p inhibitors suppressed the viability of SMC by promoting the apoptosis; miRNA-23b-3p effect was counteracted by phosphatase and tensin homolog (PTEN).
Zhang et al., 2018^39^	Human plasma	72 patients	miRNA-146a-5p	Higher miRNA-146a-5p expression is significantly associated with rupture, higher Hunt-Hess levels, and shorter overall survival.
Meeuwsen et al., 2017^40^	Human plasma	15 patients	miRNA	miRNA-183-5p were decreased in all patients, miRNA-200a-3p increased after aSAH, and miRNA-let7b-5p decreased in unruptured IA cases
Hou et al., 2017^41^	Human IA tissue, human umbilical artery SMC	8 patients	miRNA-370-3p	miRNA-370-3p was involved in the development of cerebral aneurysm by targeting Kinase Insert Domain Receptor (KDR) and blocking AKT/FOXO1 signaling pathway.
Wang et al., 2019^42^	Human IA tissue, human umbilical artery SMC	12 patients	miRNA-138, circRNA-0020397	Decreased expression of circRNA-0020397 in IA may contribute to the decreased VSMC proliferation via increasing miRNA-138 and decreasing Kinase Insert Domain Receptor (*KDR*) gene expression.
Sun et al., 2022^43^	Human plasma, human umbilical vein EC	24 patients	miRNA	miRNA-323a-3p exacerbates IA inflammation via AMPK/NF-κB signaling through AdipoR1, while AdipoR1 plasmid, AMPK activator, or si-NF-κB reduce its pro-inflammatory effects.
Jiang et al., 2022^44^	Human plasma, GEO	58 patients	miRNA	Serum miRNA-1246 levels were elevated in IA patients. Target genes of miRNA-1246, *TP53*, glycogen synthetase kinase (*GSK*), and transcription factor *YY1* may be significant for development of intracranial aneurysms. miRNA-1246 is involved in inflammation, lipid metabolism, and atherosclerosis pathways.
Jin et al., 2022^45^	Human IA tissue, human brain microvascular EC	20 patients	miRNA	miRNA-139-5p targets *FGB*, which is elevated in IA tissues while miRNA-139-5p is reduced. Increasing miRNA-139-5p or silencing FGB inhibits brain microvascular EC proliferation and tube formation and decreases α-SMA and CXCR4 levels.
Wu et al., 2021^46^	Human plasma	189 patients	miRNA	The expression levels of circulating miRNA-126 were increased unruptured IA patients and peaked on day 7 in aSAH patients before moderately decreasing by day 14.
Sun et al., 2017^47^	Human serum, human IA tissue	60 patients	miRNA	Aneurysm patients exhibit decreased miRNA-29b levels and increased autophagy markers.
Luo et al., 2016^48^	Human IA tissue	13 patients	miRNA	Dysregulation of miRNA-9 is responsible for the development of IA via targeting myocardin (*MYOCD*).
Wang et al., 2016^49^	Human serum	165 patients	miRNA	Patients with IA had significantly higher miRNA-29a expression; miRNA-29a expression was strongly associated with rupture, Hunt-Hess level, and surgical timing.
Bekelis et al., 2016^50^	Human IA tissue	7 patients	mRNA, miRNA	Patients with IA had significantly higher miRNA-29a expression; miRNA-29a were strongly associated with rupture, Hunt-Hess, and surgical timing.
Li et al., 2014^51^	Human serum	133 patients	miRNA	miRNA-16 and miRNA-25 are independent factors for IA occurrence, with adjusted odds ratios of 1.52 and 1.53, respectively.
Jiang et al., 2013^52^	Human IA tissue	14 patients	miRNA	18 miRNAs were confirmed by qPCR to be robustly downregulated in ruptured IA patients.
Cui et al., 2022^53^	Human IA tissue, rat IA model, human arterial SMC	22 patients 42 SPF rats	miRNA	The relative expression level of miRNA-29b in human IA tissue was significantly lower than in normal brain arteries. miRNA-29b possibly participated in the occurrence of intracranial aneurysms by mediating the target gene Beclin1 to regulate smooth muscle cell autophagy.
Wang et al., 2017^54^	Rat IA tissue, rat VSMC	12 patients	miRNA	miRNA-1 expression was significantly elevated in aneurysmal regions of IA rat models, correlating with aneurysm size. It directly targeted *BCL-2* and downregulated BCL-2 protein expression in cerebral VSMC. Inhibiting miRNA-1 suppressed VSMC proliferation ability and promoted apoptosis of cerebral VSMC via *BCL-2* gene regulation.
Pan et al., 2021^55^	Human IA tissue, human VSMC	30 patients	miRNA	In IA, Survival Associated Mitochondrial Melanoma-Specific Oncogenic Noncoding RNA (SAMMSON) and premature miRNA-130a were deregulated, while mature miRNA-130a was upregulated. SAMMSON was overexpressed in IA and suppressed VSMC proliferation by inhibiting miRNA-130a maturation.
Luo et al., 2022^56^	Human serum	85 patients	miRNA	Serum miRNA-126 expression was significantly lower in IA patients compared to controls (*p* < .05), and its diagnostic value for IA was high, with an AUC of 0.945.
Holcomb et al., 2015^57^	White rabbit aneurysm model	6 animals	miRNA	Three miRNAs were downregulated, and five miRNAs were upregulated, both were associated with inflammatory response, cellular migration and coagulation. ANKRD1 and TACR1 were involved in regulation of matrix metalloproteinases.
Deng et al., 2023^58^	Human brain VSMC	25 patients	miRNA	miRNA-140 was upregulated in IA patients, promoting IA progression by targeting BCL2L2 to regulate human brain VSMC apoptosis. Knockout of miRNA-140 inhibited human brain VSMC apoptosis and promoted cell proliferation.
Yue et al., 2022^59^	Human serum, human IA tissue	38 patients	miRNA	In IA tissues, circRNA-FOXO3 and KLF6 were upregulated, while miRNA-122-5p was reduced.

IA: intracranial aneurysm; VSMC: vascular smooth muscle cell; EC: endothelial cell; SMC: smooth muscle cell; aSAH: acute subarachnoid hemorrhage.

**Table 4.** Overview of studies focusing on DNA/RNA methylation, circRNA, lncRNAs, and histone modifications.

Author, year, reference	Study model	*N* of samples	Epigenetic target	Main findings
Yuan, 2023^60^	Rat brain microvascular EC	8 patients	m6A (N6-methyladenosine), RNA methylation	In IA, decreased m6A methylation, *WTAP* gene downregulation, and *ALKBH1* gene upregulation were observed. *WTAP* gene overexpression reversed tumor necrosis factor-α (TNF-α) effects on rat brain microvascular EC, suggesting it may protect against IA progression by enhancing m6A methylation.
Maimaiti, 2023^13^	Genome database (human and mouse)	73 unruptured-IA patients 83 ruptured-IA patients	DNAm	Significant DNAm pattern differences were identified between unruptured IA, ruptured IA, and normal samples, leading to the development of a predictive model for IA rupture based on DNAm-related genes.
Li, 2023^61^	GEO, ImmPort database	89 patients	DNAm	Three immune-related genes *- CXCR4, S100B,* and *OSM* - were significantly involved in IA formation and progression, with altered methylation patterns correlating with their expression: *CXCR4* and *OSM* were hypomethylated and upregulated, while *S100B* gene was hypermethylated and downregulated in IA.
Xu, 2022^62^	Human blood	22 unruptured-IA patients, 22 ruptured-IA patients	DNAm of the GSTA4 gene	IA patients exhibited decreased DNAm and increased mRNA expression of *GSTA4*, with significantly lower methylation levels in women with IA compared to controls.
Zhao, 2023^63^	Human blood, human brain VSMC	48 patients	DNAm of the MAP3K10 gene	*MAP3K10* gene methylation was significantly lower in IA patients, especially women, and was linked to blood triglyceride levels, positively in men and negatively in women.
Shafeeque, 2020^64^	Human blood	230 patients	DNAm (folate and methionine cycles)	Genetic variants in folate and methionine cycle genes (e.g., *MTHFR, MTRR, MTR, BHMT, DNMT1*) were associated with the risk of IAs by altering DNA methylation levels, potentially leading to elevated homocysteine levels, a known risk factor for IA development.
Chen, 2020^65^	Human tissue, genome database	15 (mRNA – GEO), 9 (methylation – GEO), 3 IA patients	DNAm of ADAMTS genes	IA development was linked to genetic variants, differential expression, and abnormal methylation in *ADAMTS* genes, with *ADAMTSL1* identified as a central gene in the regulatory network.
Chen, 2019^66^	Human blood	48 IA patients 22 brain AVM	DNAm of the CDKN2A gene	*CDKN2A (p16)* gene methylation levels were significantly higher in brain AVM patients, especially females, compared to IA patients and controls, suggesting it as a potential early diagnostic indicator for brain AVMs. In a normal setting, P16 protein blocks abnormal cell growth and proliferation.
Wang Z, 2021^67^	Human blood	48 patients	DNAm of the PTBP1 gene	IA patients show significant methylation differences at multiple CpG sites, with long-term tobacco exposure increasing *PTBP1* promoter methylation, lowering PTBP1 protein expression, and contributing to IA pathogenesis. No significant methylation difference was found between ruptured and unruptured IA groups.
Zhou et al., 2017^68^	Human plasma	70 IA and brain AVM patients	DNAm of the PDGFD gene	CpG1 methylation levels of the *PDGFD* gene promoter were significantly higher in IA patients and correlated with apolipoprotein E (APOE) levels.
Li et al., 2022^69^	GEO	12 patients	RNAm regulator	Principal component analysis (PCA) results showed lower m6A scores in IA than controls, with high-confidence predictions for m6A sites on six hub targets (*CDK1, ASPM, AURKB, BUB1B, MKI67*, and *TPX2*).
Maimaiti et al., 2022^70^	GEO	157 patients	RNAm regulator	Gene enrichment analysis revealed significant differences in tight junction, p53 pathway, and neurogenic locus notch homolog (NOTCH) signaling pathway varied significantly in m6A modifier patterns. Three m6A modification patterns showed significant differences in m6A regulator expression, immune microenvironment, and strong links to immune cells.
Zhou et al., 2022^71^	Human plasma, human primary artery SMC	96 patients	DNAm	*PNPLA6* methylation was higher in IA patients, especially in males and older individuals. *PNPLA6* mRNA expression decreased in IA patients. PNPLA6 protein levels were inversely correlated with elevated DNAm in participants. *PNPLA6* transcription was enhanced after treatment with a methylation inhibitor.
Wang et al., 2016^72^	Human plasma	48 patients	DNAm (NOS1AP-promoter)	AVM patients had significantly higher NOS1AP promoter DNAm levels compared to IA patients.
Laarman et al., 2018^73^	Postmortem human Circle of Willis (CoW) tissue	4 patients	DNAm, RNA	IA-associated single nucleotide peptides (SNPs) were significantly enriched in regulatory regions of the Circle of Willis, with some likely altering transcription factor binding. Nearby, 102 genes expressed in the were in proximity to these regulatory regions.
Arati et al., 2019^74^	Human blood	50 patients	DNAm (APOE), F13A)	Apolipoprotein E promoter methylation showed no significant association with aSAH risk or patient outcomes. *F13A* promoter methylation status was significantly associated with aSAH risk in males, but not with post-aSAH outcomes.
Xu, 2023^75^	Human IA tissue, human VSMC	30 patients	circRNA-ATL1 and miRNA-455	In IA patients, circRNA-ATL1 was upregulated, and miRNA-455 was downregulated. Silencing circRNA-ATL1 inhibited VSMC proliferation, migration, and phenotypic changes, targeting *SIRT5* and miRNA-455.
Ding, 2021^76^	Human brain VSMC	N/A	circRNA-DOCK1, miRNA-409-3p, and myeloid cell leukemia sequence 1 (MCL1).	Hydrogen peroxide exposure reduced proliferation and increased apoptosis in human brain VSMC, while decreasing circRNA-DOCK1 expression. Overexpressing circRNA-DOCK1 countered these effects and reduced apoptosis. miRNA-409-3p, upregulated by hydrogen peroxide, was targeted by circRNA-DOCK1.
Qin, 2021^77^	Human IA tissue, human umbilical artery SMC	21 patients	circRNA-ARFIP2, miRNA-338-3p, and kinase insert domain receptor (KDR)	circRNA-ARFIP2, downregulated in IA tissues, promotes VSMC proliferation, migration, and invasion by sponging miRNA-338-3p and upregulating *KDR*. miRNA-338-3p, overexpressed in IA, negatively regulates *KDR. KDR* is crucial for VSMC functions, acting downstream of both circRNA-ARFIP2 and miRNA-338-3p.
Yin K and Liu X, 2021^78^	Human IA tissue, human VSMC	cell culture	circRNA-0020397, miRNA-502-5p and Gremlin 1 (GREM1)	circRNA-0020397 or GREM1 protein expression was decreased in VSMCs isolated from IA patients. miRNA-502-5p overexpression suppressed VSMC viability and reduced PCNA level, while these effects were attenuated by GREM1 upregulation.
Wang et al., 2022^79^	Human IA tissue, human umbilical artery SMC	32 patients	circRNA	In IA tissues and human umbilical artery SMC, circRNA-0021001 and Gremlin 1 (GREM1) expression levels were increased, while miRNA-148b-3p was decreased. circRNA-0021001 served as a sponge of miRNA-148b-3p to regulate GREM1 expression also could repress proliferation ability and induce apoptosis of human umbilical artery SMC
Zhang et al., 2022^80^	Human IA tissue, human umbilical artery SMC	38 patients	circRNA	In IA patients, circLIFR and KDR were downregulated, while miRNA-1299 was upregulated. circLIFR promotes human umbilical artery SMC proliferation, migration, and invasion by targeting miRNA-1299 and inhibiting apoptosis.
Zhu 2022^81^	GEO	51 patients	Ferroptosis-related genes, lncRNAs, miRNAs, mRNA	A ferroptosis-related ceRNA network with significant iron deposition in IA tissues was identified, featuring key regulatory networks like PVT1-hsa-miRNA-4644-SLC39A14 and DUXAP8-hsa-miRNA-378e/378f-SLC2A3.
Chu et al., 2020^82^	Human umbilical vein EC, human IA tissue	40 patients	lncRNA-AC007362, miRNA-493, monocyte chemoattractant protein-1 (MCP-1)	Under shear stress, AC007362 expression increased due to reduced promoter DNA methylation, while MCP-1 expression increased by sponging miRNA-493.
Lv et al., 2021^83^	Human IA tissue	82 patients	lncRNA	In IA, especially ruptured IA, NORAD and KDM1A were upregulated, while miRNA-136-5p was downregulated. NORAD promoted VSMC proliferation and migration by sponging miRNA-136-5p, which targets *KDM1A,* and these effects are reversed by miRNA-136-5p upregulation or *KDM1A* knockdown.
Li et al., 2017^84^	Human IA tissue	12 patients	lncRNAs, mRNAs, miRNAs	In aneurysm cases 1150 lncRNAs, 2545 mRNAs, and 286 miRNAs were differentially expressed, and a ceRNA network of 8401 miRNA-lncRNA-mRNA interactions was constructed.
Hu et al., 2022^85^	Human IA tissue, human VSMC	20 patients	lncRNAs, mRNAs, miRNAs	ANRIL was downregulated in the plasma and arterial wall tissues; overexpression of ANRIL promoted VSMC proliferation and inhibited apoptosis by directly targeting miRNA-7, which regulates FGF2 expression.
Poppenberg, 2021^5^	Genome-based	16 IA-associated single nucleotide peptides (SNPs)	Histone marks: H3K4me1, H3K27ac, H3K9ac	Enhancer marks on IA risk haplotypes were significantly enriched in endothelial cells (ECs) and fibroblasts, but not in smooth muscle or immune cells. Bioinformatics analysis showed that genes within TADs containing these regions are associated with extracellular matrix components and enzymatic activity, and most histone-marked TADs are involved.
Sun, 2023^86^	Human IA tissue, human vascular EC	75 patients	HDAC9, miRNA-34a-5p, and vascular endothelial growth factor-A (VEGFA)	HDAC9 was upregulated in IA, causing vascular EC injury by repressing miRNA-34a-5p via histone deacetylation, which subsequently increases VEGFA expression.
Cai et al., 2021^87^	Human IA tissue, IA rat model	90 patients, 100 Sprague-Dawley rats for IA modeling	Histone deacetylases 9, miRNA-92a, Bcl-2-like protein 11	HDAC9 inhibition upregulated miRNA-92a, which represses IA progression by silencing *BCL2L11*.
Poppenberg, 2019^12^	Genome database	Not mentioned	Histone modifications (H3K4me1 and H3K27ac), DNAm, and transcription factor binding sites (CTCF).	Genetic risk loci for IA were enriched with histone marks and transcription factor binding sites in endothelial cells, suggesting IA risk likely operates through endothelial dysfunction.

IA: intracranial aneurysm; VSMC: vascular smooth muscle cell; EC: endothelial cell; SMC: smooth muscle cell; AVM: arteriovenous malformation; NORAD: non-coding RNA activated by DNA damage; TAD: topologically associating domain; GEO: Gene Expression Omnibus database.

**Table 5.** Epigenetic mechanisms associated with aneurysm formation, inhibition, and rupture.

Mechanism	“Pro-aneurysmal”	“Contra-aneurysmal”	Rupture-related
miRNA	miRNA-1, miRNA-9, miRNA-15a-5p, miRNA-16, miRNA-23b-3p, miRNA-25, miRNA-26a, miRNA-26b, miRNA-26b-5p, miRNA-29a, miRNA-29b, miRNA-34a, miRNA-34a-5p, miRNA-143, miRNA-145, miRNA-140, miRNA-155-5p, miRNA-191-3p, miRNA-199a, miRNA-205, miRNA-323a-3p, miRNA-335-5p, miRNA-365, miRNA-370-3p, miRNA-374a-5p, miRNA-423-5p, miRNA-424-5p, miRNA-425-3p, miRNA-448-3p, miRNA-4735-3p, miRNA-497, miRNA-1246, miRNA-130a, SAMMSON	miRNA-331-3p, miRNA-4735-3p, miRNA-124-5p, miRNA-144-5p, miRNA-21, miRNA-29-3p, miRNA-448-3p, miRNA-138, miRNA-139-5p, miRNA-29b, miRNA-122-5p	miRNA-155, miRNA-144-5p, miRNA-143/145, miRNA-486-5p, miRNA-146a-5p, miRNA-200a-3p, miRNA-let7b-5p, miRNA-29a
Methylation	CXCR4, OSM, GSTA4, MAP3K10, ADAMTS, PTBP1, PNPLA6 (all via hypomethylation)	WTAP, S100B, m6A, PNPLA6	F13A and PDGFD promoter methylation
circRNA	circRNA-ATL1, circRNA-DOCK1, circRNA-ARFIP2, circRNA-0020397, circLIFR	–	–
lncRNA	lncRNAs of ceRNA network, lncRNA-AC007362, NORAD (via KDM1A)	ANRIL (via VSMC proliferation)	NORAD
Histone modifications	H3K4me1, H3K27ac, H3K9ac, HDAC9	–	–

VSMC: vascular smooth muscle cell; NORAD: non-coding RNA activated by DNA damage.

### microRNAs

MicroRNAs are the most studied targets due to their crucial role in gene regulation, remarkable stability in biological fluids, high evolutionary conservation across species, and consequent therapeutic potential. In this review, 46 papers with 38 unique microRNA targets were identified; microRNA-143, microRNA-29a, and microRNA-155 were the most frequently studied. These studies have investigated aneurysm association, formation, inhibition, and/or rupture. Key findings are summarized in Table 3, which includes the targets, study models, and primary results.^14–59^

Over half of the identified microRNA targets are associated with formation or progression of intracranial aneurysms. For example, microRNA-16, microRNA-143, microRNA-200, microRNA-26a and microRNA-29a, and microRNA-448-3p were significantly upregulated in IA patients compared to the controls, with microRNA-200 and microRNA-143 showing particularly high levels in patients with multiple or ruptured aneurysms.^14,15^ On the other hand, several microRNAs have been identified as “protective” against the formation and progression of intracranial aneurysms, including miRNA-331-3p, miRNA-4735-3p, miRNA-124-5p, miRNA-144-5p, miRNA-21, miRNA-29-3p, miRNA-448-3p, miRNA-138, miRNA-139-5p, miRNA-29b, and miRNA-122-5p. For example, microRNA-331-3p inhibited IA formation and progression by downregulating TNF-α and CD14, thus maintaining the contractile phenotype of VSMCs and reducing VSMC apoptosis.^16^

Finally, some microRNAs have been found to be associated with both promoting and inhibiting aneurysm formation, depending on the context. MicroRNA-513b-5p downregulation in ruptured aneurysms (RA) and unruptured aneurysms (UA) targeted *COL1A1* and *COL1A2* genes, regulating RIP1-RIP3-MLKL and MMP pathways to enhance cell death and apoptosis.^23^

### Circular RNAs

CircRNA is a type of non-coding RNA molecule that forms a closed loop structure through a covalent bond between its 3′ and 5′ ends, providing high stability. CircRNAs have been shown to modulate microRNA activity, transcription, and protein interactions, and exhibit tissue-specific expression. These characteristics make circRNAs potential biomarkers with high utility. Six studies explored the association and/or role of circular RNAs, including circRNA-ATL1, circRNA-DOCK1, circRNA-ARFIP2, circRNA-0020397, and circLIFR, in IA formation^42,75–80^

These studies utilized various experimental models, including human tissue samples from IA patients and controls and in vitro models using human vascular smooth muscle cells (VSMCs). These studies consistently demonstrated that circRNAs play crucial roles in regulating VSMC function, which is central to IA pathogenesis. Specifically, most identified circRNAs (circ-ATL1, circ_DOCK1, circ-ARFIP2, circ_0020397, and circLIFR) were shown to modulate VSMC proliferation, migration, and/or invasion.^42,75–78^^,80^ Additionally, circ_DOCK1 and circLIFR were found to attenuate apoptosis, while circ_0021001 silencing induced apoptosis in VSMCs. Several studies identified important downstream targets of circRNA-miRNA axes, including *SIRT5, MCL1, KDR*, and *GREM1*, which are involved in various cellular processes relevant to IA formation.^42,75–78^^,80^

### Long non-coding RNAs

Five studies investigated the role of lncRNAs in IAs.^81–85^ The studies revealed several mechanisms and pathways involving lncRNAs: competing endogenous RNA (ceRNA) networks and miRNA–lncRNA–mRNA interactions,^81,84^ the ANRIL/miR-7/FGF2 pathway,^85^ the NORAD/miR-136-5p/KDM1A axis,^83^ and the AC007362/miR-493/MCP-1 pathway.^82^ The identified lncRNAs were associated with various functional changes in VSMCs and endothelial cells.

Li et al.^84^ found that mRNAs were involved in muscle contraction and vascular smooth muscle contraction, providing insights into mechanisms of IA formation. Similarly, Zhu et al.^81^ conducted bioinformatics analysis on public datasets to investigate ferroptosis-related genes and non-coding RNAs in IAs. They identified key regulatory pathways and validated their findings by showing significant iron deposition in IA tissues, suggesting the role of ferroptosis in IA pathogenesis.

Chu et al.^82^ studied lncRNA AC007362 in human IA samples and endothelial cells, discovering that shear stress increased AC007362 expression due to reduced promoter DNA methylation. It was found that AC007362 sponged miR-493, increasing MCP-1 expression, suggesting that altered hemodynamics might contribute to IA formation via epigenetic regulation. Similarly, Hu et al.^85^ investigated the role of lncRNA ANRIL in IAs, finding lncRNA ANRIL downregulation in IA patients. They demonstrated in vitro that ANRIL promotes vascular smooth muscle cell proliferation and inhibits apoptosis via the ANRIL/miR-7/FGF2 pathway, suggesting therapeutic potential for IA. Studies have shown that the presence of certain lncRNAs is associated with aneurysm rupture. Lv et al.^83^ used RNA-seq to study lncRNAs in IA, finding upregulated lncRNAs NORAD and KDM1A, and downregulated miR-136-5p expression in IA. These results are more pronounced in ruptured IA versus unruptured IA. It was proposed that NORAD acts as a competing endogenous RNA for miR-136-5p, upregulating KDM1A and affecting VSMC phenotype regulation in IA formation and rupture.

### Histone modifications

Histone modifications have emerged as critical regulators in the pathophysiology of intracranial aneurysms (IAs), with a growing body of evidence highlighting their role in aneurysm growth and rupture. Four studies^5,12,86,87^ (see Table 3) have explored the association of specific histone modifications, including H3K4me1 (methylation of histone H3), H3K27ac, H3K9ac (acetylations of histone H3), and the epigenetic factor HDAC9, with IAs.

Poppenberg et al.^12^ identified genetic risk loci enriched with histone marks and transcription factor binding sites in endothelial cells, linking these modifications to endothelial dysfunction, a key driver of aneurysm formation. In 2021, Poppenberg's team^5^ further analyzed IA-associated SNPs and histone marks, including H3K4me1, H3K27ac, and H3K9ac, uncovering significant enrichment of enhancer histone marks in endothelial cells and fibroblasts. These findings emphasize the epigenetic regulation of genes within topologically associating domains (TADs), many of which are differentially expressed in IA tissues. HDAC9 has been identified as a pivotal factor in IA development. Cai et al.^87^ demonstrated that inhibiting HDAC9 in a rat IA model and human IA samples upregulates miR-92a, which represses IA progression by silencing BCL2L11. Conversely, Sun et al.^86^ found that HDAC9 upregulation in IA tissues promotes vascular endothelial cell injury by repressing miR-34a-5p. This repression leads to increased VEGFA expression, resulting in endothelial cell proliferation, enhanced migration, suppressed apoptosis, and increased vascular permeability, all processes critical to aneurysm growth and rupture.

### DNA and RNA methylation

In this review, the roles of DNA and RNA methylation in the pathogenesis of IAs were studied in 16 papers.^13,60–74^ Several studies have shown that the methylation status of specific genes is associated with aneurysm rupture. For example, methylation status of *F13A* gene^74^ and the *PDGFD* gene promoter^68^ were found to be associated with the aneurysm rupture. DNA methylation patterns show significant differences between unruptured IA, ruptured IA, and normal samples, leading to the development of predictive models for IA rupture risk.^13^

Hypomethylation of several genes, including *CXCR4*, *OSM*, *GSTA4*, *MAP3K10*, *ADAMTS*, *PTBP1*, and *PNPLA6*,^61–63^^,65,67,71^ has been identified as being associated with the aneurysm formation. In contrast, aneurysm inhibition was found to be associated with the methylation changes in *WTAP*, *S100B*, *m6A*, and *PNPLA6* genes.^60,61,71^ Several studies have highlighted that decreased N6-methyladenosine (m6A) methylation levels, including the downregulation of *WTAP* and upregulation of *ALKBH1* gene expression, may play a protective role against IA progression.^60,69^ Gender-specific differences in methylation patterns have been observed, including lower *MAP3K10* methylation in women^63^ and higher *CDKN2A* methylation in female patients with brain arteriovenous malformations compared to IA patients and controls.^66^ The observed gender-specific differences and the impact of genetic variants further emphasize the need for personalized approaches in understanding and treating IA, with methylation emerging as a potential biomarker and therapeutic target for future research.

## Discussion

### Epigenetics definition

Epigenetic changes are regulatory processes that modulate gene expression without altering the primary DNA sequence. We have chosen five of the most extensively studied epigenetic mechanisms (DNA methylation, histone acetylation, lncRNAs, miRNAs, and circRNAs) while newer mechanisms such as histone ubiquitination, SUMOylation, and ADP-Ribosylation and others have only recently emerged and represent promising avenues for future research. Among the five mechanisms studied, histone acetylation (loosens chromatin to promote transcription), lncRNAs (serve as scaffolds or guides to enhance gene regulation), and circRNAs (act as miRNA sponges to protect gene expression) primarily support transcription. In contrast, DNA methylation (silences gene promoters) and miRNAs (bind target mRNAs to block translation or promote degradation) are generally associated with transcriptional suppression.

### Research rationale

Recent advancements in sequencing technologies, reduced costs, and growing insights from genetic research have enabled neurosurgeons to approach their most challenging diseases (such as intracranial aneurysms [IAs]) with new precision. Epigenetic mechanisms have emerged as a key research focus in the context of intracranial aneurysms (IAs) due to their involvement in essential biological processes (such as inflammation, vascular smooth muscle cell proliferation, and extracellular matrix remodeling) that are fundamental to aneurysm development and rupture.^5,44,63,75,80^

If successfully validated, epigenetic targets may serve as biomarkers or therapeutic targets for IA diagnosis, disease screening, and personalized treatment solutions. Established modifiable risk factors such as smoking, hypertension, and age are well known to be strongly associated with aneurysm formation, growth, and rupture.^88–91^ While emerging work has identified DNA methylation, histone modifications, and non-coding RNAs as potential epigenetic contributors,^13,92,93^ direct comparisons between these mechanisms and traditional clinical risk factors are lacking. If epigenetic pathways are more clearly defined, a potential advantage would be the ability to pharmacologically mitigate smoking- or hypertension-related vascular injury without relying solely on behavioral modification.

Several pharmacological classes have been investigated for their potential to modulate epigenetic mechanisms in intracranial aneurysms. The most robust aneurysm-specific evidence comes from SIRT1 activators, where resveratrol reduced rupture rates in a mouse model by upregulating Sirt1 and downregulating NF-κB-mediated inflammation.^94^ Other categories remain more speculative. DNMT inhibitors such as 5-aza-2′-deoxycytidine have been proposed on the basis of DNA methylation changes identified in aneurysm tissue,^62^ although direct therapeutic data are lacking. HDAC inhibitors, including valproic acid and trichostatin A, have shown anti-inflammatory and neuroprotective effects in central nervous system models;^95^ however, findings from thoracic aortic aneurysm models indicate that broad HDAC inhibition may increase aneurysm susceptibility,^96^ cautioning against direct extrapolation. Finally, miRNA-based strategies remain of interest, as several dysregulated miRNAs (notably miR-126) have been linked to intracranial aneurysm pathophysiology and rupture risk, highlighting potential diagnostic and therapeutic applications.^97^ Moreover, a recent bioinformatics study by Lai et al.^98^ highlighted the potential involvement of estrogen receptor signaling (ESR1), suggesting that hormonal regulation may also contribute to IA pathophysiology and represent an additional therapeutic target.

Epigenetic changes, along with genetic alterations, can be detected in liquid biopsy samples (such as blood, saliva, or urine) enabling the development of non-invasive diagnostic and monitoring tools based on distinct molecular signatures.^99,100^ Epigenetic mechanisms represent an exceptionally prominent focus in intracranial aneurysm research, as evidenced by the fact that 80.5% of relevant publications have been produced in the last five years (2018–2023), with Chinese groups contributing greatly, comprising 66 (83.6%) papers in this review.

### Current landscape and outlook

The study by Boga^15^ reported significant upregulation of miRNA-448-3p in aneurysm tissues compared to normal vascular tissues, while Zhang et al.^34^ showed that miRNA-448-3p is associated with reduced IA progression through modulation of macrophage-driven inflammation. Rather than representing conflicting evidence, these findings may reflect the multifaceted role of miRNAs as components of complex epigenetic networks. It is possible that the observed upregulation of miRNA-448-3p represents a secondary or compensatory response to ongoing inflammation within aneurysmal tissue, rather than a direct driver of aneurysm formation. Therefore, such outcomes should be interpreted with caution in the context of underlying pathophysiological mechanisms.

Of the 79 papers analyzed, there were only three instances of overlapping targets identified: circRNA-0020397,^42,78^ miRNA-143/145,^14,35,36,50^ and miRNA-29b.^27,29,47,53^ The limited overlap in identified targets across studies, as observed in our analysis, reflects a broader challenge in genetic and epigenetic research, one driven by variability in sample types, sequencing strategies, data processing pipelines, and cohort characteristics. Similar inconsistencies have been reported in other fields of genomics and are often attributed to insufficient standardization, underpowered study designs, and population heterogeneity. To address this, researchers have proposed several solutions, including harmonized protocols, meta-analyses, and cross-validation using multi-cohort datasets.^101,102^

More recently, AI-based integrative approaches and machine learning algorithms have shown promise in identifying robust patterns across heterogeneous datasets, helping to uncover signature genes despite methodological diversity, particularly in contexts such as abdominal aortic aneurysm growth^103^ or intracranial aneurysm (IA) rupture.^104^

### Limitations

This review provides a thorough and current summary of the existing evidence on the role of epigenetics in the pathophysiology of IA. However, it is not without its limitations. The studies included in this review show significant variability in both the types of samples and the methodologies employed to investigate the epigenetic mechanisms underlying intracranial aneurysms (IAs). Researchers utilized a wide range of human tissue samples, including those from IA patients, normal arterial tissue, and various vascular cell types such as vascular smooth muscle cells (VSMCs) and endothelial cells or serum. Furthermore, several studies incorporated animal models, particularly rats, to simulate IA conditions, while others relied on bioinformatics analyses using publicly available datasets to supplement experimental data. In addition, many of the manuscripts show associations between the targets and outcomes (aneurysm formation, inhibition, and rupture), rather than causality, highlighting the need for further experimental studies to demonstrate causal relationships.

## Conclusion

Epigenetic mechanisms will likely continue to be a central focus in intracranial aneurysm (IA) research due to their potential to unravel the intricate and multifactorial nature of this condition. Unlike genetic mutations, which are relatively stable, epigenetic changes are dynamic and can be modulated by environmental factors, lifestyle choices, and various disease states. This dynamic nature makes epigenetic mechanisms a critical link between genetic predisposition and environmental influences in the pathogenesis and progression of IAs. Moreover, the reversible nature of epigenetic modifications presents a promising avenue for the development of novel therapeutic interventions. By specifically targeting these epigenetic alterations, future research may pave the way for strategies aimed at preventing aneurysm formation, stabilizing existing aneurysms, or reducing the risk of rupture.

## Abbreviations

ANRIL: antisense non-coding RNA in the INK4 locus
aSAH: acute subarachnoid hemorrhage
AVM: arteriovenous malformation
circRNA: circular RNA
DNAm: DNA methylation
EC: endothelial cell
GEO: Gene Expression Omnibus database
IA: intracranial aneurysm
miRNA: microRNA
mRNA: messenger RNA
MMP: matrix metalloproteinase
NORAD: non-coding RNA activated by DNA damage
SMC: smooth muscle cell
TAD: topologically associating domain
TGF: transforming growth factor
TNF: tumor necrosis factor
VSMC: vascular smooth muscle cell.
